# PNMA5 Promotes Bone Metastasis of Non-small-Cell Lung Cancer as a Target of BMP2 Signaling

**DOI:** 10.3389/fcell.2021.678931

**Published:** 2021-05-31

**Authors:** Fei Huang, Yaqiang Cao, Caihong Wang, Ruilong Lan, Bing Wu, Xianhe Xie, Jinsheng Hong, Lengxi Fu, Gui Wu

**Affiliations:** ^1^Central Lab, The First Affiliated Hospital, Fujian Medical University, Fuzhou, China; ^2^Fujian Key Laboratory of Precision Medicine for Cancer, The First Affiliated Hospital, Fujian Medical University, Fuzhou, China; ^3^Key Laboratory of Radiation Biology of Fujian Higher Education Institutions, The First Affiliated Hospital, Fujian Medical University, Fuzhou, China; ^4^CAS Key Laboratory of Computational Biology, CAS-MPG Partner Institute for Computational Biology, Shanghai Institute of Nutrition and Health, Shanghai Institutes for Biological Sciences, University of Chinese Academy of Sciences, Chinese Academy of Sciences, Shanghai, China; ^5^Department of Radiation Oncology, The First Affiliated Hospital, Fujian Medical University, Fuzhou, China; ^6^Department of Chemotherapy, The First Affiliated Hospital, Fujian Medical University, Fuzhou, China; ^7^Department of Orthopedics, The First Affiliated Hospital, Fujian Medical University, Fuzhou, China

**Keywords:** PNMA5, NSCLC, bone metastases, BMP2, target gene

## Abstract

Bone metastases frequently occur in NSCLC patients at the late stage, indicating poor survival. However, mechanisms about the initiation of NSCLC bone metastases remain largely unclear. In our previous reports, BMP2 signaling activation has been found to enhance NSCLC bone metastases through enhancing carcinoma cells migration, invasion, osteoclasts differentiation and osteoblasts immature differentiation. Nevertheless, downstream target genes of BMP2 contributing to those processes still remain unknown. In this project, we find that the expression of *Pnma5* is higher in metastatic bone tumors of Lewis lung carcinoma than in metastatic lung tumors and parental Lewis lung cells. *Pnma5* overexpression not only can promote cell migration and invasion of NSCLC cells but also tumor-induced osteoclasts differentiation. Interestingly, knockdown of *Pnma5* in Lewis lung cells blocks BMP2 signaling from inducing Lewis lung cells migration and invasion. Although BMP2 signaling can promote Lewis lung cells-induced osteoclasts differentiation from macrophages, this effect can also be blocked when *Pnma5* is knocked down in Lewis lung cells. Moreover, *Pnma5* can promote NSCLC bone metastases *in vivo* as the downstream target of BMP2. Those results above indicate that BMP2 signaling enhances NSCLC bone metastases via its direct downstream target gene *Pnma5*. This research reveals the detailed molecular mechanism about how BMP2 signaling contributes to NSCLC bone metastases via PNMA5 and provides a new potential therapeutic target for the treatment of NSCLC bone metastases.

## Introduction

Lung cancer is one of the most deadly cancers worldwide (Hanahan and Weinberg, 2011; Vargas and Harris, 2016). Non-small-cell lung cancer (NSCLC) is the most common type of lung cancer. Nearly 80% of lung cancer cases are NSCLC, with subtypes of adenocarcinoma, squamous carcinoma, adenosquamous carcinoma, large cell carcinoma and sarcomatoid carcinoma (Herbst et al., 2018). When NSCLC patients were at late stages, distant metastasis frequently occurs, resulting in poor prognosis. The median survival time is between 14 and 17 months when distant metastases occur in NSCLC patients (Juan and Popat, 2017). Thus, the therapy for metastatic NSCLC is still challengeable.

Several cancer types tend to cause bone metastases, such as breast cancer, prostate cancer and NSCLC (McAllister and Weinberg, 2014). About 30–40% of NSCLC patients accompany with bone metastases at late stages. Moreover, among NSCLC patients with bone metastases, lung adenocarcinomas are more commonly seen than squamous cancers (Santini et al., 2015; Oliveira et al., 2016). Previous reports have revealed some mechanisms presenting in NSCLC bone metastases (Popper, 2016; Sathiakumar et al., 2017). Parathyroid hormone-related peptide (PTHrP) and receptor activator of Nuclear Factor-kappaB ligand (RANKL), which play key roles in osteoclasts activation (Popper, 2016; Macedo et al., 2017), are found to have functions in NSCLC bone metastases (Nakamura et al., 2006; Kuo et al., 2013). Moreover, miRNA-33a, which targets PTHrP, has been reported to reduce bone metastatic burden in NSCLC (Kuo et al., 2013). In addition, matrix metalloproteinase (MMPs), are supposed to be involved in bone metastases (McAllister and Weinberg, 2014; Wood et al., 2014; Massague and Obenauf, 2016; Ganguly et al., 2020; Zhang et al., 2020), as they contribute to extracellular matrix degradation and interaction between cancer cells with stroma. Thus, signaling pathways that activate MMPs, like transforming growth factor (TGF-β), Wnt, CXCR4, and NFκB, may also play roles in NSCLC bone metastases via MMPs (Popper, 2016). However, in comparison with breast cancer and prostate cancer, the detailed mechanisms about how NSCLC bone metastasis occurs remain largely unclear (McAllister and Weinberg, 2014).

The high expression of bone morphogenic protein 2 (BMP2) has been reported in NSCLC (Langenfeld et al., 2003, 2005; Bieniasz et al., 2009; Choi et al., 2012; Fei et al., 2013). BMP2 signaling activation can enhance lung adenocarcinoma cell proliferation, migration, invasion and lung metastases (Langenfeld et al., 2003; Hsu et al., 2011; Chu et al., 2014). Moreover, BMP2 in the tumor microenvironment is also associated with NSCLC prognosis. High expression of BMP2 in the stroma may result in poor prognosis in NSCLC (Rajski et al., 2015). In our previous reports, BMP2 signaling is found to enhance NSCLC bone metastases via both osteolytic and osteoblastic mechanisms (Huang et al., 2020). However, the downstream target genes of BMP2 signaling associated with NSCLC bone metastases still remain unknown.

PNMA5 is a member of the Paraneoplastic Ma (PNMA) family. Members of the PNMA family have been reported to play roles in carcinoma development (Pang et al., 2018). Lee et al. (2016) has demonstrated that PNMA5 is highly expressed in colon cancer and can enhance the apoptosis of breast cancer cells *in vitro*. According to our RNA-seq data in the previous report (Huang et al., 2020), the expression of *Pnma5* is found to be higher in bone metastatic tumors of Lewis lung carcinoma than in lung metastatic tumors and parental Lewis lung cells. However, researches on the roles that PNMA5 play in NSCLC are rare.

In this project, we have found that PNMA5 can enhance the migration and invasion of NSCLC cells. In addition, PNMA5 can enhance the NSCLC-induced osteoclasts differentiation to promote bone metastasis of NSCLC. Interestingly, PNMA5 is found to be a downstream target of BMP2 signaling in NSCLC. BMP2 signaling enhances bone metastasis of NSCLC via PNMA5. Altogether, PNMA5 can be a downstream target of BMP2 signaling and plays roles in NSCLC bone metastases. Furthermore, PNMA5 can be a new potential therapeutic target for NSCLC patients with bone metastases at late stages.

## Materials and Methods

### Antibodies, siRNAs, and Reagents

Antibodies used in this study: anti-PNMA5 (Abcam, ab150921); monoclonal anti-Smad1/5 (Cell Signaling Technology, 6944) and anti-β-Actin (Sigma, A1978). The sequences of siRNAs applied to knock down *Pnma5* in LLC cells were as follows. SiRNA 1# Target: GCAGAAACCTTATGTTAGA; siRNA 2# Target: CTAGAAATGATCCCAACAA; siRNA 3# Target: CTGACTACTTGCTACGTTT. Reagents: BMP2 (R&D, 355-BM-100); Tris-HCl, NaCl and other reagents were purchased from Sigma.

### Mice

Female C57BL/6 mice (6–8 weeks of age) were used in this study, and they were bred and maintained in a specific pathogen-free animal facility at Fujian Medical University. Mice were euthanized with carbon dioxide asphyxiation at the end of observation. All animal experiments were approved by the Animal Ethical Committee of Fujian Medical University (2018-039).

### Cells and Transfection

NSCLC cell lines: A549 (ATCC number: CCL-185) and Lewis lung cells (ATCC number: CRL-1642). The macrophage cell line: Raw 264.7(ATCC number: TIB-71). Lewis lung cells were cultured in RMPI1640 (Invitrogen, Carlsbad, CA, United States) which contained 10% fetal bovine serum (FBS) (Hyclone, Utah, United States); A549 and Raw 264.7 cells were cultured in DMEM (Invitrogen) with 10% FBS (Hyclone). Transfection of siRNAs into LLC and A549 cells were performed by using polyethylenimine (polysciences, Inc., PA, United States).

### Lewis Lung Carcinoma Metastasis

For the tail veins injection model: 1 × 10^6^ LLCs were injected into the tail veins of per C57/BL6 mice to make the lung and bone metastatic model. For the orthotopic model: 1 × 10^6^ LLCs were pre-treated with the vehicle or 20 ng/mL BMP2 for 24 h. Then the tumor cells were injected into the left lung lobes of per C57BL/6 mice to make the orthotopic model. LLCs frequently tend to colonize in the lungs and bones. Mice were sacrificed with carbon dioxide asphyxiation and tumor tissues were harvested for further analyses after 35 days of injection. Bone metastatic tumor sizes were measured by tumor length and width by using clipper directly and lung metastatic tumor sizes were measured via the HE staining photos. The tumor volumes were calculated via the formula V = (L × W × W)/2, where V is tumor volume, W is tumor width, L is tumor length (Huang et al., 2020).

### Hematoxylin and Eosin Stain

The tissue sections were treated as what mentioned in the previous report (Huang et al., 2020). The sections were cut into 2.5 μm tissue sections after they were embedded in paraffin. Tissue sections were then dewaxed with xylene. 100, −95, and −75% alcohol gradients were used to rehydrate the sections. The tissue sections were stained in Hematoxylin for 20 min, and then differentiated with 1% hydrochloric acid for 30 s. After that, the sections should go through 15 min of PBS blue staining and 3 min of eosin staining. Sections were dehydrated with a gradient of 95–100% alcohol after rinsing. The sections were cleaned with xylene for two times, before the sections were finally mounted with a neutral resin. Photos were taken by Olympus microscope BX53.

### Immunoblotting

This assay was conducted as what mentioned in the previous report (Huang et al., 2020). The TNE buffer (10 mM Tris-HCl, 150 mM NaCl, 1 mM EDTA, 0.5% NP40, pH = 7.5) were used to lyse cells and tissues. Then, the cell lysates were mixed with 4 × loading buffer (40 mM Tris-HCl, 200 mM DTT, 4% SDS, 40% Glycerol, 0.032% Bromophenol Blue, pH = 8.0). The samples were run with 4% stacking gel and 10% separating gels. After that, proteins on the gels were transferred to nitrocellulose filter membranes. And then, antibodies were incubated. The membranes’ exposure was done with thermo Pierce ECL and FluorChem E (Protein Simple).

### Cell Migration Assays

Cells were treated as what mentioned in the previous report (Huang et al., 2020). Cells with density of 1 × 10^4^ cells/insert were seeded on the upper layer of Corning cell culture insert with polycarbonate membrane (Transwell@, 8.0μm pore size, Corning) and cultured in media without FBS. The complete culture media (10% FBS) with or without 20 ng/mL BMP2 were placed below the cell permeable membrane in the well plates. After an incubation for 24 h in 37°C, 5% CO2, the cells migrating through the membrane were stained with 0.1% crystal violet and counted.

### Cell Invasion Assays

Cells were treated as what mentioned in the previous report (Huang et al., 2020). 10:1 DMEM and matrigels (BD BioSciences) were utilized to pre-treat Corning cell culture insert with polycarbonate membrane (Transwell@, 8.0 μm pore size, Corning). Cells with density of 1 × 10^5^ cells/insert were seeded on the pre-treated inserts and cultured in media without FBS. The complete culture media (10% FBS) with or without 20 ng/mL BMP2 were filled in the well plates below the cell permeable membrane. After incubated for 48 h in 37°C, 5% CO_2_, the cells migrating through the membrane were stained with 0.1% crystal violet and counted.

### Cell Proliferation Assays

Cells were treated as what mentioned in the previous report (Huang et al., 2019). Cells were seeded in 96 well plates at a density of about 3,000 cells/well. Relative cell intensity was measured with the cell counting kit-8 (CCK-8, Dojindo Molecular Technologies) after indicated time.

### Tartrate-Resistant Acid Phosphatase (Trap) Staining

3 × 10^4^ murine pre-osteoclast RAW 264.7 cells were seeded directly into each well of the 6-well co-culture plates, 3 × 10^4^ lung cancer cells were seeded into each of the Corning cell culture inserts with polycarbonate membrane (Transwell^@^, 0.4 μm pore size) of the co-culture 6-well plates in triplicate. Lung cancer cells were treated with the vehicle or 20 ng/mL BMP2. The culture media were DMEM medium supplemented with 10% FBS and changed every 2 days. TRAP staining was performed on day 6 with a leukocyte acid phosphatase kit (Sigma, 387A). TRAP^+^ cells were scored as mature osteoclasts and quantified (Huang et al., 2020).

### Chromatin Immunoprecipitation Assay

Cells were treated as what mentioned in the previous report (Huang et al., 2019). After Lewis lung cells and A549 cells were cross-linked by 1% formaldehyde solution for 15 min, the cells were neutralized with 125 mM Glysine for 5 min. Then, cells were lysed using lysis buffer (1% SDS, 50 mM Tris pH 8.0, 5 mM EDTA, proteinase inhibitors). Subsequently, the cell lysates were sonicated to get DNA fragments (300–500 bp). The sonicated lysates were pre-absorbed with protein A beads for 30 min at 4°C and then incubated with 10 μg antibodies (control IgG and anti-HIF1α) overnight at 4°C. At Day 2, Protein A agarose beads were added into the cell lysates to bind the antibodies and targeted proteins for 3 h at 4°C. After the bound beads were washed four times sequentially with salt buffers, the bound immunocomplexes were eluted from the beads with elution buffer (25 mM Tris, pH 8.0, 10 mM EDTA, 0.5% SDS) by heating at 65°C for 15 min. 1 mg/mL protease K was used for reversing the crosslinking at 65°C overnight. The obtained DNAs were purified and subjected to quantitative real-time PCR. Sequences of ChIP primers were as follows. Mouse *Pnma5* promoter: Forward 5′- CAGGGATTAAAGATGTGC -3′, Reverse 5′- GAGTAGGATAGGGCAGAG -3′. Human PNMA5 promoter: Forward 5′- TCAGCCTTCAGAAACATG -3′, Reverse 5′- CAAAGTGCTGGGATTAGA-3′.

### Quantitative Real-Time PCR

This assay was conducted as what mentioned in the previous report (Huang et al., 2019). Total cell RNA was extracted with TRIzol (Invitrogen). Then, cDNA was synthesized via reverse transcripts with Revertra Ace (Promega, Madison, United States). Quantitative real-time PCR was performed with an ABI QuantStudio 5 system. The results were measured by the comparative Ct method. The relative expression values of targeted genes were normalized to GAPDH expression. The primer sequences were as follows. Mouse *Pnma5*: Forward 5′- GTGGTTGTCAAACCCCGTAG-3′, Reverse 5′-TTCCCTGTAGGAACAGTGCTAA-3′; human PNMA5: Forward 5′-AGATGAGGGCCGAAGTATGAC′, Reverse 5′-GCTCTAAAGGTGGGGATCTAACT-3′; Mouse and human Gapdh: Forward 5′-CATGGCCTTCCGTGTTCCTA-3′, Reverse 5′-CCTGCTTCACCACCTTCTTGAT-3′.

### Statistical Analysis

The Student’s *t*-test, one-way ANOVA test, Wilcox rank sum test and log-rank test were used as indicated in the figure legends. *P* < 0.05 were considered statistically significant.

## Results

### *Pnma5* Is Highly Expressed in Bone Metastasis of Lewis Lung Carcinoma

**FIGURE 1:**
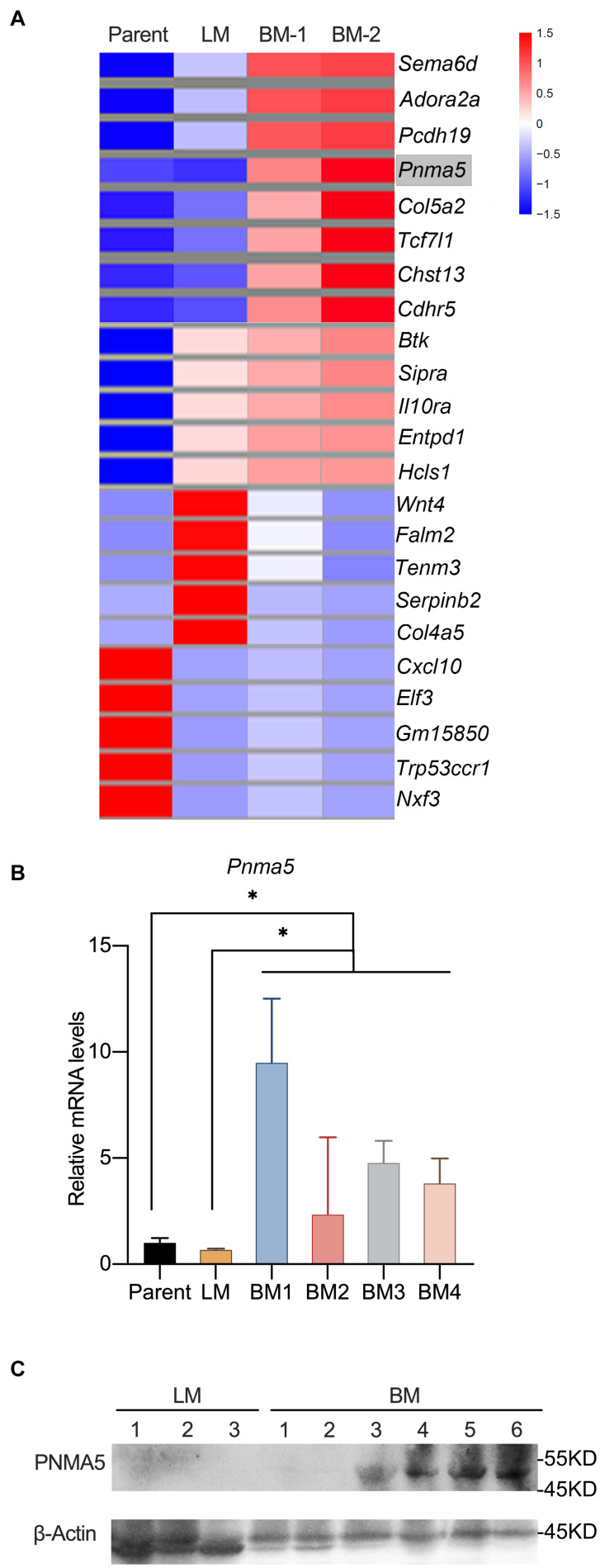
*Pnma5* is highly expressed in bone metastasis of Lewis lung carcinoma. **(A)** Heatmap with mRNA-based expression of representative genes in bone metastasis (BM-1 and BM-2), lung metastasis (LM) and parental Lewis lung cells (Parent). The gene names were shown on the right of the heatmap. Values are normalized intensities, log2. **(B)** Comparison of relative *Pnma5* mRNA levels of bone metastasis (BM), lung metastasis (LM) and parental Lewis lung cells (Parent) by qPCR. *P < 0.05. **(C)** Lysates of the indicated tissues were harvested to be subjected to western blot for PNMA5. β-Actin was the reference for the blots. LM, lung metastasis; BM, bone metastasis.

Lewis lung carcinoma originated from a C57BL/6 mouse was a spontaneous lung adenocarcinoma (Bertram and Janik, 1980; Zhu et al., 2018). Tail veins injection of carcinoma cells could be a classical way to make animal models of tumor metastasis (Brady et al., 2016; Lu et al., 2019; Wang et al., 2020). Lewis lung carcinoma cells (LLCs) were injected into tail veins of C57BL/6 mice to establish lung metastases and bone metastases models. In our previous reports, RNA-seq was carried out to analyze the transcriptome differences among metastatic bone tumors, metastatic lung tumors and parental Lewis lung cells (Huang et al., 2020). The results shown here were based upon data assessed online at the Gene Expression Omnibus (GEO) (NO. GSE148101). Some representative differential expressed genes were shown in Figure 1A. We majorly focused on the significant differential expressed genes (DEGs) which were expressed higher in metastatic bone tumors than in metastatic lung tumors and parental cells, because those genes were more likely to contribute to bone metastasis of Lewis lung carcinoma. Consistently, MMPs with high expression in metastatic bone tumors based on our RNA-Seq data had been reported to play roles in prostate or breast cancer bone metastasis (Larson et al., 2013; Colden et al., 2017; Ganguly et al., 2020; Zhang et al., 2020). *Pnma5* was another gene that was expressed higher in metastatic bone tumors than in metastatic lung tumors and parental cells (Figure 1A). Although, functions of *Pnma5* in cancer progression had been partially revealed in colon cancer and breast cancer, the roles of *Pnma5* playing in NSCLC bone metastases were still unclear (Lee et al., 2016). Thus, we went further to research whether *Pnma5* promoted NSCLC bone metastases. We examined the mRNA levels of *Pnma5* in four metastatic bone tumors, one metastatic lung tumor and parental cells by quantitative real-time PCR. In consistence with the RNA-seq data, the expression of *Pnma5* was also higher in bone metastases than in lung metastases and parental cells (Figure 1B). Besides, the protein levels of *Pnma5* were also increased in metastatic bone tumors of Lewis lung carcinoma in contrast with metastatic lung tumors (Figure 1C).

### BMP2 Signaling Activation Induced the Expression of *Pnma5*

**FIGURE 2:**
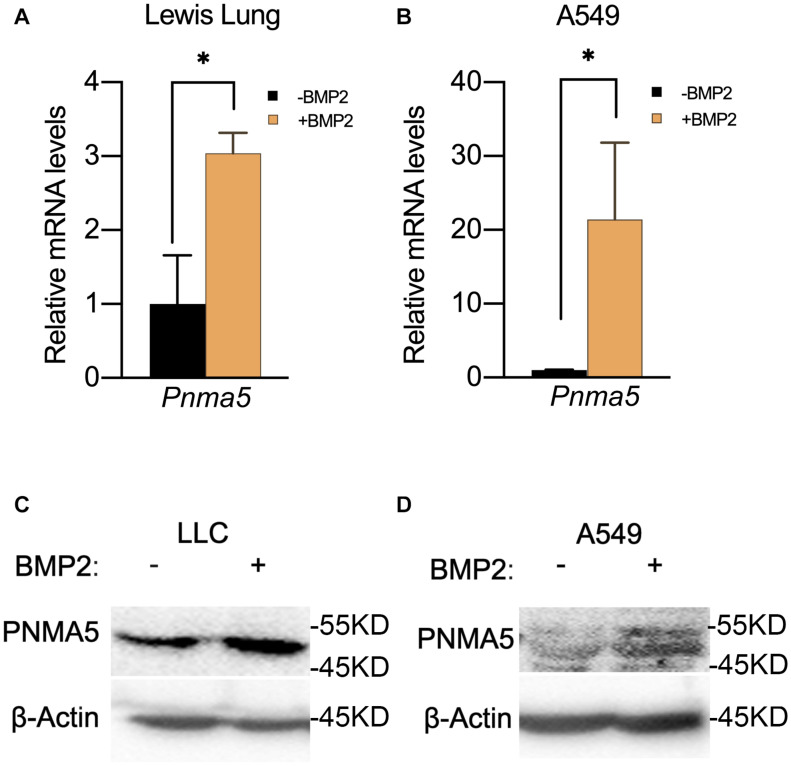
BMP2 signaling activation induced the expression of PNMA5. **(A)** Comparison of relative *Pnma5* mRNA levels of LLC cells with or without BMP2 treatment by qPCR. *P < 0.05. **(B)** Comparison of relative *Pnma5* mRNA levels of A549 cells with or without BMP2 treatment by qPCR. *P < 0.05. **(C)** LLC cells treated with or without BMP2 for 1 h. Lysates of the indicated treated LLC cells were harvested to be subjected to western blot for PNMA5. β-Actin was the reference for all the blots. **(D)** A549 cells treated with or without BMP2 for 1 h. Lysates of the indicated treated A549 cells were harvested to be subjected to western blot for PNMA5. β-Actin was the reference for all the blots.

In our previous report, BMP signaling was activated in bone metastatic tumors of NSCLC and BMP2 enhanced bone metastases of NSCLC (Huang et al., 2020). Thus, we proposed the hypothesis that BMP2 signaling activation could induce the expression of *Pnma5* in NSCLC cells. Interestingly, we found that BMP2 treatment could induce the upregulation of *Pnma5* mRNAs in both mice NSCLC cells LLCs and human NSCLC cells A549 (Figures 2A,B). Moreover, the protein levels of PNMA5 were increased when mice NSCLC cells LLCs and human NSCLC cells A549 were under BMP2 treatment (Figures 2C,D).

### *Pnma5* Was the Direct Target of BMP2 Signaling

**FIGURE 3:**
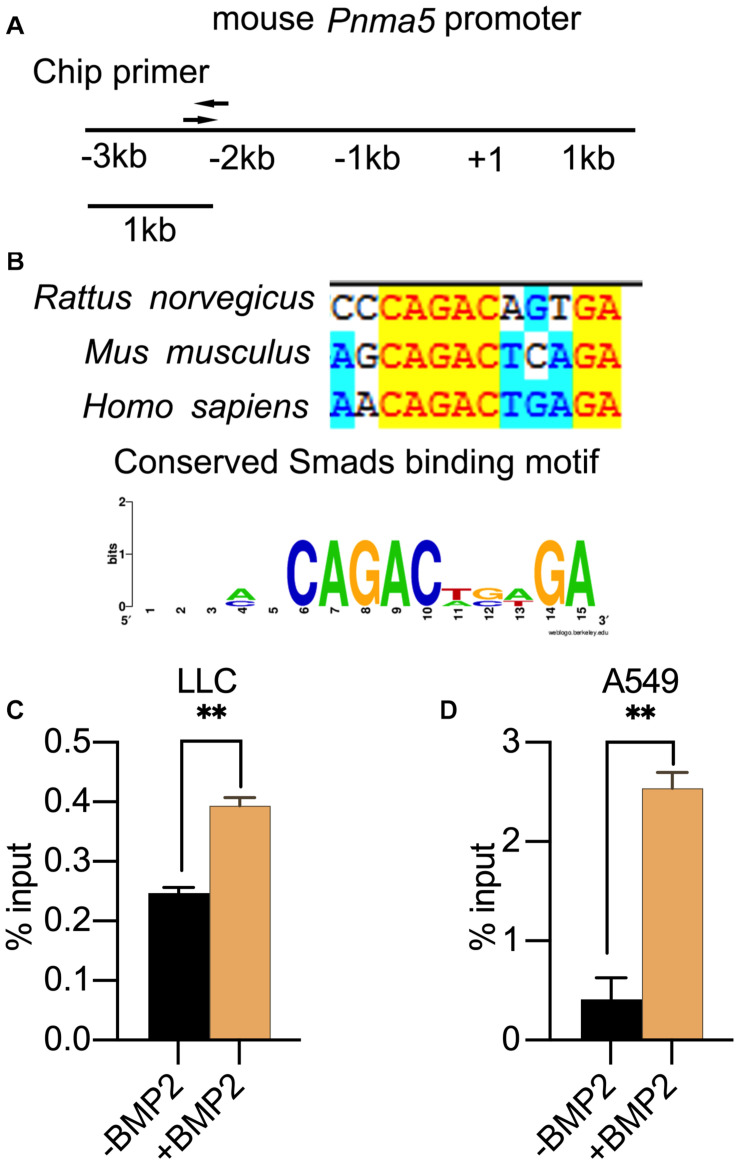
PNMA5 was the direct target of BMP2 signaling. **(A)** The DNA sites where the ChIP primers were designed at the PNMA5 promoter. **(B)** The “CAGAC” element in the PNMA5 promote is conserved across species. The “CAGAC” Smad1/5 binding motif across species was analyzed by WebLogo. **(C)** ChIP assay was performed in LLC cells treated with or without BMP2 for 6 h using the Smad1/5 antibodies. The immunoprecipitated DNA was amplified by quantitative real-time PCR with the primers designed in **(A)** **P < 0.01. **(D)** ChIP assay was performed in A549 cells treated with or without BMP2 for 6 h using Smad1/5 antibodies. The immunoprecipitated DNA was amplified by quantitative real-time PCR with the primers designed in **(A)** **P < 0.01.

When BMP2 binds to its receptor BMPRII and BMPRI, Smad1/5/8 can be phosphorylated by BMPRI and then translocated into nucleus with Smad4 to regulate the expression of downstream target genes (Miyazono et al., 2005; Wu et al., 2016). The Smad1/5/8 usually binds to the motif “CAGAC” or “GGCGCC” (Smads binding element sequences, SBEs) in the promoter of its target genes. Therefore, we analyzed the PNMA5 promoter sequences to find the “CAGAC” or “GGCGCC” motifs. We found several SBEs in the −3 kb∼ + 1 bp region of mice and human PNMA5 (Figure 3A). Interestingly, one of the SBEs was highly conserved in human, mice and rats (Figure 3B). To make sure whether Smad1/5/8 could bind this SBE of *Pnma5* promoter, we further conducted the ChIP-qPCR assay in LLC and A549 cells with the Smad1/5 antibody. Primers were designed on the conserved SBE region along the *Pnma5* promoter (Figure 3A). As the amplicon covered the conserved SBE in the promoter of *Pnma5*, we showed that BMP2 signaling activation could lead to the binding of Smad1/5 to SBE in LLC and A549 cells (Figures 3C,D). Thus, these data demonstrated that Smad1/5 bound to the SBE of *Pnma5* promoter to contribute to the upregulation of *Pnma5* when BMP2 signaling was activated. *Pnma5* was a direct target gene of BMP2 signaling.

### BMP2 Signaling Induced the Migration and Invasion of NSCLC Cells via PNMA5

**FIGURE 4:**
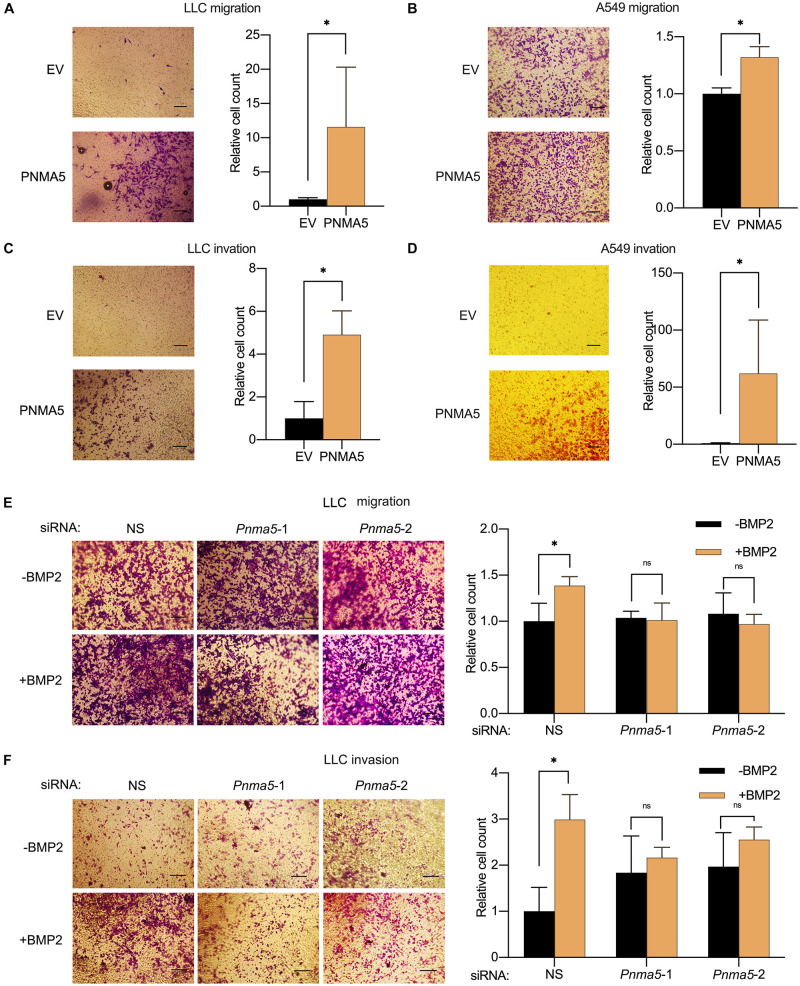
BMP2 signaling induced the migration and invasion of NSCLC cells via PNMA5. **(A,B)** The 1 × 10^4^ empty vector (EV) and PNMA5 overexpressed LLC **(A)** or A549 **(B)** cells were seeded and cultured in media without FBS on the upper layer of the Corning cell culture insert with polycarbonate membrane (Transwell@, 8.0μm pore size) for 24 h. The complete culture media were placed below the cell permeable membrane in the well plate. The migrating cells were stained with crystal violet and were taken photos. Average cell numbers of at least three fields were counted and shown on the right. *P < 0.05. **(C,D)** Corning cell culture insert with polycarbonate membrane (Transwell@, 8.0μm pore size) were pre-treated with 10:1 DMEM and matrigels (BD BioSciences). The 1 × 10^5^ empty vector (EV) and PNMA5 overexpressed LLC **(C)** or A549 **(D)** cells were seeded and cultured in media without FBS on the upper layer of the pre-treated cell culture insert for 24 h. The complete culture media were placed below the cell permeable membrane in the well plate. The invading cells were stained with crystal violet and were taken photos. Average cell numbers of at least three fields were counted and shown on the right. *P < 0.05. **(E)** The 1 × 10^4^ siRNA scrambler, siRNA *Pnma5*-1 and siRNA *Pnma5*-2 expressed LLC cells were treated as described in **(A)**. The migrating cells were stained with crystal violet and were taken photos. Average cell numbers of at least three fields were counted and shown on the right. *P < 0.05. **(F)** The 1 × 10^5^ siRNA scrambler, siRNA *Pnma5*-1 and siRNA *Pnma5*-2 expressed LLC cells were treated as described in **(C)**. The invading cells were stained with crystal violet and were taken photos. Average cell numbers of at least three fields were counted and shown on the right. *P < 0.05.

In our previous report, BMP2 signaling activation enhanced NSCLC cells migration and invasion (Huang et al., 2020). And PNMA5 was confirmed to be a direct target of BMP2 signaling in NSCLC cells based on results above. Thus, we further examined the role of PNMA5 played in migration and invasion of NSCLC cells by the transwell assay. We overexpressed PNMA5 in LLC cells and A549 cells (Supplementary Figures 1A,C). Interestingly, PNMA5 overexpression enhanced migration and invasion of LLC cells and A549 cells (Figures 4A–D). We further focused on whether BMP2 signaling promoted migration and invasion of NSCLC cells via PNMA5. The expression of *Pnma5* was knocked down in LLC cells (Supplementary Figure 1B). We found that BMP2 could no longer induce the migration and invasion of LLC cells with low expression of *Pnma5*, indicating that BMP2 signaling enhanced LLC cells’ migration and invasion via *Pnma5* (Figures 4E,F).

### BMP2 Signaling Mediated NSCLC-Induced Osteoclasts Differentiation via PNMA5

**FIGURE 5:**
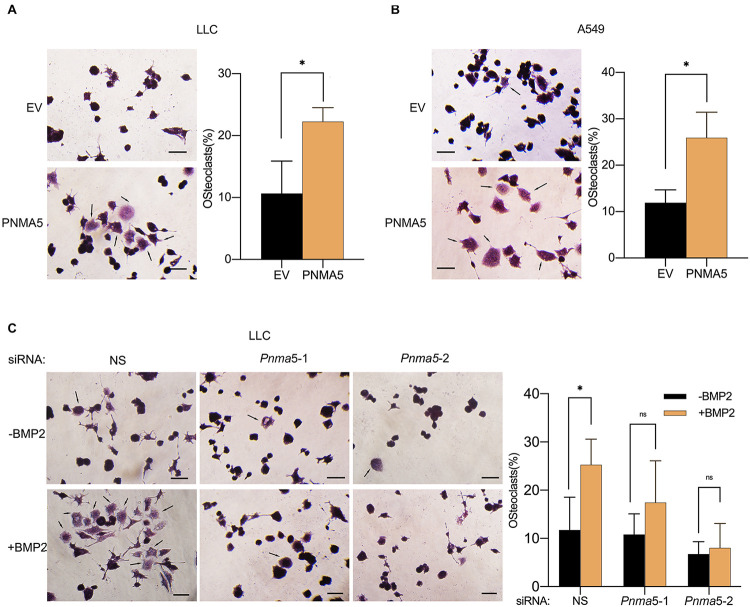
BMP2 signaling enhanced NSCLC cells-induced osteoclasts differentiation via PNMA5. **(A,B)** 3 × 10^4^ empty vector (EV) and PNMA5 overexpressed LLC **(A)** or A549 **(B)** cells were seeded into the Corning Cell Culture Inserts with polycarbonate membrane (Transwell@, 0.4 μm pore size) and 3 × 10^4^ RAW 264.7 cells were seeded below the polycarbonate membrane into the wells of the 6-well co-culture plates (Corning). TRAP staining was conducted for RAW 264.7 cells cultured for 6 days by a leukocyte acid phosphatase kit. Representative photos were shown. Scale bars, 50μM. Average Trap^+^ cell numbers of at least three fields were calculated and shown on the right. Black arrows showed the osteoclasts. The *p*-value was based on the Student’s *t*-test. *P < 0.05. **(C)** 3 × 10^4^ siRNA scrambler, siRNA *Pnma5*-1 and siRNA *Pnma5*-2 expressed LLC cells were seeded into the Corning Cell Culture Inserts with polycarbonate membrane (Transwell@, 0.4μm pore size) and 3 × 10^4^ RAW 264.7 cells were seeded directly into the wells of the 6-well co-culture plates (Corning). TRAP staining was conducted for RAW 264.7 cells cultured for 6 days by a leukocyte acid phosphatase kit. Representative photos were shown. Scale bars, 50μM. Average Trap^+^ cell numbers of at least three fields were calculated and shown on the right. Black arrows showed the osteoclast. The *p*-value was based on the Student’s *t*-test. *P < 0.05.

Bone metastases can be classified into osteolytic, osteoblastic or mixed subtypes based on the effect of cancers on normal bone remodeling (Selvaggi and Scagliotti, 2005; Quayle et al., 2015). The osteolytic mechanism has been reported to be associated with NSCLC bone metastases (Nakamura et al., 2006; Kuo et al., 2013). In osteolytic metastases, osteoclasts played an important role in the remodeling of bones (Mundy, 2002; Li et al., 2019; Brunetti et al., 2020). Interestingly, in our previous reports, BMP2 had been shown to stimulate NSCLC-induced osteoclast differentiation from macrophages (Huang et al., 2020). Thus, we went further to examined whether BMP2 signaling mediated NSCLC-induced osteoclasts differentiation via PNMA5. As shown in Figures 5A,B, overexpression of PNMA5 in NSCLC cells promoted the tumor-induced osteoclast differentiation of macrophages. Furthermore, if *Pnma5* was knocked down in LLC cells, BMP2 could not mediate NSCLC-induced osteoclast differentiation from macrophages (Figure 5C). The results above indicated that BMP2 signaling mediated NSCLC-induced osteoclasts differentiation via PNMA5.

### BMP2 Signaling Enhanced Bone Metastases of Lewis Lung Carcinoma via PNMA5 *in vivo*

**FIGURE 6:**
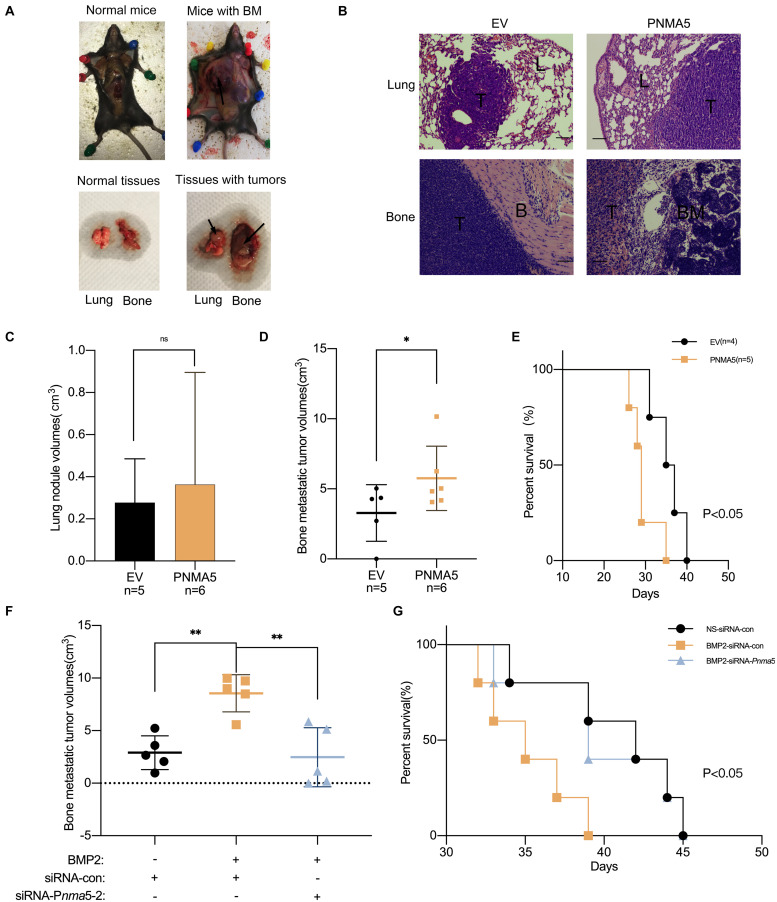
BMP2 signaling enhanced bone metastasis of Lewis lung carcinoma via PNMA5 *in vivo*. **(A)** 1 × 10^6^ empty vector (EV) and PNMA5 overexpressed LLC cells were injected into the left lung lobes of C57BL/6 mice. Graphs of tumor-burdened mice were shown. Black arrows showed the bone metastatic lesions. Normal and tumor burdened lung and bone tissues were shown. Black arrows showed the metastatic lesions. BM: metastatic bone tumors. **(B)** Representative HE staining of tissues from lungs and bones in **(A)**. Scale bars of the 100× photos were 100 μM. L, normal lung tissues; T, tumor tissues; B, normal bone tissues; BM, normal bone marrow tissues. **(C)** Tumor sizes of mice lungs in **(A)** were measured and tumor volumes were calculated. **(D)** Tumor sizes of mice bones in **(A)** were measured and tumor volumes were calculated. *P < 0.05. **(E)** Death of mice in **(A)** were recorded and survival curves were drawn. *P < 0.05. **(F)** 1 × 10^6^ siRNA scrambler, siRNA *Pnma5*-1 and siRNA *Pnma5*-2 expressed LLC cells were injected into the left lung lobes of C57BL/6 mice. Tumor sizes of mice bones in **(A)** were measured and tumor volumes were calculated. **P < 0.01. **(G)** Death of mice in **(F)** were recorded and survival curves were drawn. *P < 0.05.

Cell migration, invasion and osteolysis are all key factors in bone metastases of carcinoma (Labelle et al., 2011; Macedo et al., 2017). According to the results above, PNMA5 enhanced the migration and invasion of NSCLC cells as a target gene of BMP2 signaling. It promoted NSCLC-induced osteoclasts differentiation via PNMA5 as well. Thus, we further focused on whether BMP2 signaling promoted NSCLC bone metastases via PNMA5 *in vivo*. LLCs were injected into the left lung lobes of C57BL/6 mice. We found that LLC cells could localize in the left lungs to form the primary tumors, but also the left shoulders to form bone metastatic lesions (Figure 6A). We transfected LLC cells with the empty vector or the PNMA5-carried vector to establish the stable empty vector or PNMA5 expressed LLC cell lines. After that, the EV overexpressed or PNMA5 overexpressed LLC cells were injected into the left lung lobes of C57BL/6 mice. We found that the EV overexpressed or PNMA5 overexpressed LLC cells could both localize in the left lungs and left shoulders of 57BL/6 mice (Figure 6B). There was no difference between the average volumes of primary tumors in left lungs of the EV group and the PNMA5 group (Figure 6C). However, the average volumes of bone metastatic tumors of the PNMA5 group were much larger than that of the EV group (Figure 6D). Besides, the metastatic lesions formed in the PNMA5 group showed a more invasive phenotype in contrast with the EV group (Figure 6B), as the bone destruction occurred in the PNMA5 group but not the EV group. Furthermore, mice in the PNMA5 group survived shorter than mice in the EV group (Figure 6E). Those results above indicated that PNMA5 promoted bone metastases of LLC cells *in vivo*. In our previous reports, BMP2 was shown to enhance the bone metastases of LLC cells *in vivo* directly (Huang et al., 2020). LLC cells were pre-treated with the vehicle or 20 ng/mL BMP2 for 24 h. After that, the pre-treated LLCs were injected into the left lung lobes of C57BL/6 mice. We found that BMP2 treated LLC cells could form larger bone metastatic lesions than the vehicle treated LLC cells (Figure 6F). However, if *Pnma5* was knocked down in LLC cells, BMP2 could no longer enhance the formation of bone metastatic lesions of LLC cells (Figure 6F). The same results also could be found when the survival curve of the mice was analyzed. BMP2 treated LLC cells injected mice could survive shorter than the vehicle treated LLC cells injected mice. However, if *Pnma5* was knocked down in BMP2 treated LLC cells, the mice could survive as long as the vehicle treated LLC cells injected mice (Figure 6G). Altogether, BMP2 signaling was observed to enhance bone metastases of Lewis lung carcinoma via PNMA5 *in vivo*.

## Discussion

Bone metastases frequently occur in NSCLC, resulting in poor prognosis (Kuchuk et al., 2013; Santini et al., 2015; Oliveira et al., 2016). As the destruction of bones mediated by osteoclasts plays key roles in the formation of bone metastatic lesions, denosumab targeting RANKL (the important factor for osteoclasts differentiation) has been used to treat patients with bone metastases in clinics (D’Antonio et al., 2014; De Castro et al., 2015; Nasser et al., 2019). However, the therapy efficacy is limited. Thus, research on mechanisms about how NSCLC bone metastases occur has been a hot spot in recent years. LncRNA MALAT1, miRNA-33, CXCR4, and TGF-β signaling have all been reported to contribute to bone metastases of NSCLC (Liu et al., 2016; Popper, 2016; Liao et al., 2018; Yang et al., 2019). Interestingly, in our present work, we firstly report that PNMA5 enhances NSCLC bone metastases as a target gene of BMP2 signaling. PNMA5 maybe a new potential therapeutic target for NSCLC bone metastases treatment.

PNMA5 belongs to the PNMA protein family. The aberrant protein expression level and mutations of PNMA family members are associated with the human Paraneoplastic Disorder (PND). PND patients usually exhibit syndrome consisting of auto-immunity, neuro-degeneration, and cancer (Pang et al., 2018). High-throughput sequencing analysis reveals that PNMA5 may be associated with metastasis of colon cancer (Zhou et al., 2019). Moreover, PNMA5 has been reported to promote apoptosis in human cancers (Lee et al., 2016). However, there is still no direct evidence to show that PNMA5 contributes to cancer metastases. We have found that overexpression of PNMA5 can enhances migration and invasion of NSCLC cells but inhibits proliferation of NSCLC cells *in vitro* (Supplementary Figures 2A,B). Besides, high expression of PNMA5 enhances tumor-induced osteoclasts differentiation *in vitro* and promotes bone metastatic lesions formation *in vivo*. Our research provides direct evidence to show that PNMA5 contributes to NSCLC bone metastases rather than NSCLC cells growth. Moreover, we still observed a phenomenon, in which the migration and invasive ability of LLC cells seemed to be slightly increased but not significantly, when *Pnma5* was knocked down in LLC cells without BMP2 treatment. However, overexpression of PNMA5 could enhance the migration and invasion of LLCs. There are two potential reasons for this phenomenon. On one hand, when the expression of PNMA5 is at relative low levels, its effect on the migration and invasion of LLCs is not significant. On the other hand, PNMA5 could inhibit the proliferation of LLCs, thus knock-down of PNMA5 may increase the proliferation of LLCs which lead to the enhancement of migration and invasion of cells that we observed.

Osteoclastgenesis plays dominant roles in carcinoma bone metastases. When osteoclast activity is increased and osteoblast activity is decreased, bone resorption occurs to provide microenvironment for tumor colonization (Mathis et al., 2018). Cancer cells frequently regulate the osteoclast differentiation when bone metastases occur through secreting the key factors for osteoclastgenesis, like RANKL and PTHrP (Fornetti et al., 2018). Thus, cancer cells have been found to affect the osteoclast differentiation by some mechanisms. Previous experimental research has reported that low expression of SOSTDC1 in NSCLC cells promoted cancer cells-induced osteoclast differentiation (Chen et al., 2018). The gene expression changes of NSCLC cells can mediate the secreting factors of NSCLC cells, which subsequently regulate the osteoclast differentiation. High expression of CXCR4 in NSCLC cells have been shown to promote cancer cells-mediated osteoclast differentiation through secreting VCAM1 (Liao et al., 2018). Moreover, C5aR1 can also enhance NSCLC cells to induce osteoclast differentiation through secreting CXCL16 (Ajona et al., 2018). In recent years, exosomes, which can mediate the osteoclast differentiation, have also been found to be secreted by NSCLC cells (Taverna et al., 2017). In our research, we verified that BMP2 signaling activation could promotes LLC cells-induced osteoclast differentiation, and this effect could be blocked by knockdown of *Pnma5*. PNMA5 can be the downstream target of BMP2 signaling in mediating tumor associated osteoclasgenesis. Nevertheless, the downstream cytokines or exosomes of PNMA5 in NSCLC cells, which affect osteoclastgenesis still remains unclear, which needs further research.

In our previous work, we reported that BMP2 signaling activation could enhance NSCLC bone metastases. In the current research, we initially demonstrate that *Pnma5* is the downstream target gene of BMP2 signaling to enhance NSCLC bone metastases. There is no previous report showing the upstream signaling pathway that regulates the expression of PNMA5 until now. Thus, our work finds a new potential mechanism about how BMP2 signaling functions in regulating cancer metastases. However, the associated proteins of PNMA5 in regulating the NSCLC bone metastases still remain unknown, which needs further research in the future.

## Data Availability Statement

The datasets presented in this study can be found in online repositories. The names of the repository/repositories and accession number(s) can be found in the article/Supplementary Material.

## Ethics Statement

The animal study was reviewed and approved by the Animal Ethical Committee of Fujian Medical University (2018-039).

## Author Contributions

FH: conceptualization, data curation, formal analysis, funding acquisition, roles, and writing–original draft. GW: data curation, formal analysis, and writing–review and editing. YC: conceptualization, data curation, and formal analysis. CW, RL, BW, XX, JH, and LF: investigation. All authors read and approved the final manuscript.

## Conflict of Interest

The authors declare that the research was conducted in the absence of any commercial or financial relationships that could be construed as a potential conflict of interest.
