# Supplementary material for: PNMA5 Promotes Bone Metastasis of Non-small-Cell Lung Cancer as a Target of BMP2 Signaling
Source: Front Cell Dev Biol. 2021 May 31;9:678931. doi: 10.3389/fcell.2021.678931 (PMC8200676; doi:10.3389/fcell.2021.678931)
Supplement: Supplementary file 1 [file Data_Sheet_1.docx]

Supplementary Material

# Supplementary Data


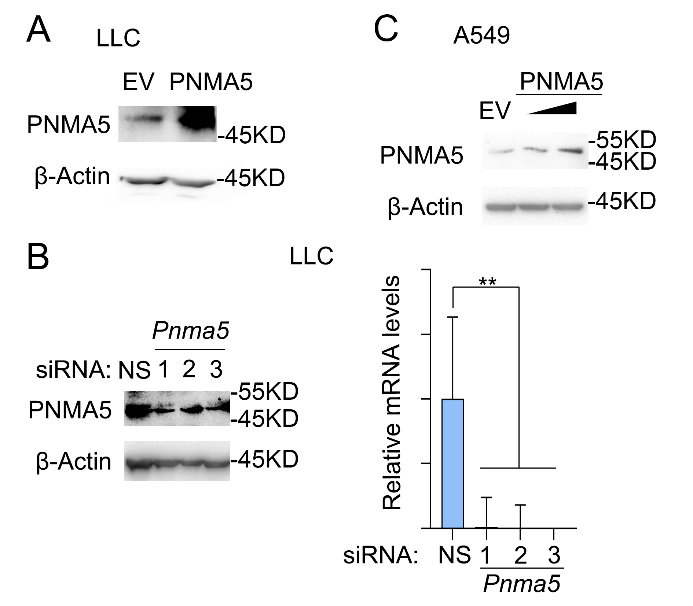


**Sup 1. The expression of PNMA5 in empty vector (EV) and PNMA5 overexpressed and siRNA scrambler, siRNA *Pnma5*-1 and siRNA *Pnma5*-2 expressed cells.**

Cell lysates of the indicated LLC cells (A, B left) and A549 (C) cells were harvested to be subjected to western blot for PNMA5. β-Actin was the reference for all the blots. Comparison of relative *Pnma5* mRNA levels of indicated LLC cells by qPCR (B right). ***: P<0.01*.


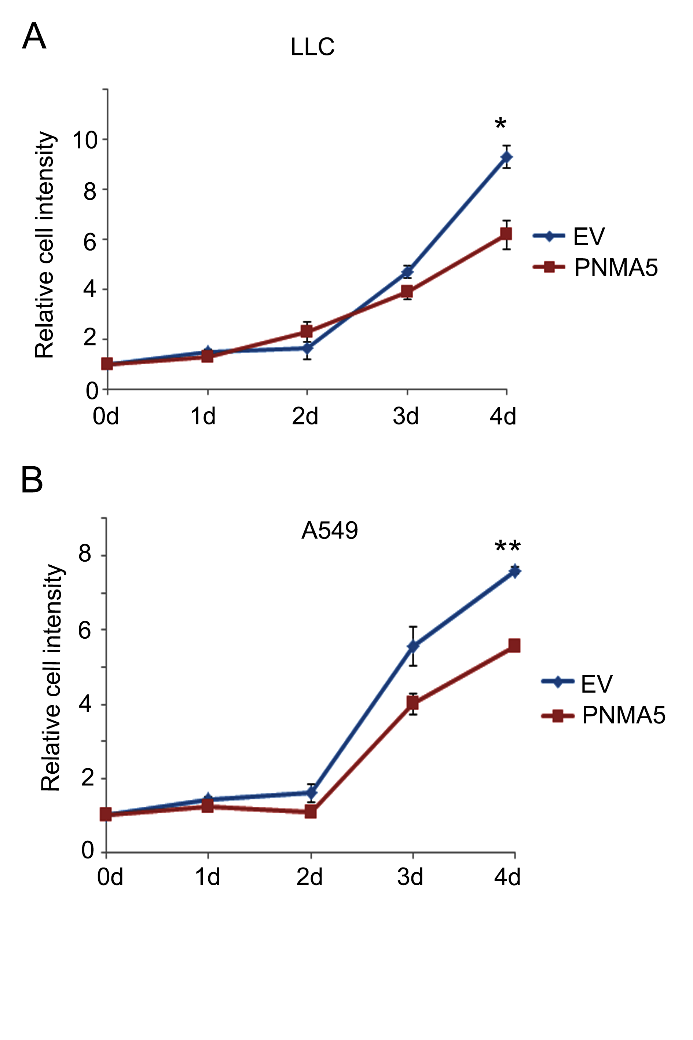


**Sup 2. PNMA5 inhibited cell proliferation of NSCLC cells.**

(A-B) About 3,000 empty vector (EV) and PNMA5 LLC or A549 cells were cultured in each well of 96 well plates. Relative cell intensity was measured with a cell-counting kit after indicated time. **: P<0.05, **: P<0.01*
